# 33 Strengthening the Infection Prevention Workforce: Evaluation of the TEPHI Infection Prevention and Control 300 Series

**DOI:** 10.1017/ash.2026.10475

**Published:** 2026-06-23

**Authors:** Sri Pulaparthi, AC Burke, Cindy Prins

**Affiliations:** 1 University of Central Florida College of Medicine; 2 RB Health Partners, Inc; 3 University of Central Florida

## Abstract

**Background:** On April 1, 2024, the U.S. Centers for Medicare and Medicaid Services mandated the implementation of Enhanced Barrier Precautions (EBP) in all nursing homes. EBP, which are intended to reduce MDRO transmission while avoiding the adverse effects associated with prolonged use of contact precautions, require the use of gowns and gloves during high-contact resident care activities for individuals with a history of MDRO colonization or infection, non-healing wounds, or indwelling medical devices. While EBP may offer important infection prevention benefits, concerns exist about increased costs, staff workload, and potential resident stigmatization. To date, there are no published data examining infection preventionists’ perceptions of EBP, and few studies have looked at methods of EBP implementation. **Methods:** To address these gaps, this study assessed experiences, challenges, and perceptions related to EBP implementation in nursing homes across Florida (UCF IRB 00007601). From May through September 2025, individuals with responsibility for infection control and EBP in their facilities were invited to take a cross-sectional, 42-item Qualtrics survey. The survey invitation was mailed to 578 free-standing nursing homes in Florida with 60 or more beds, and 90 responses were received (15.6% response rate). **Results:** Among the demographic results, the majority (96.7%) of respondents worked in infection control, with most (45.3%) having 1 to 3 years of experience as an infection preventionist and approximately 25% holding a certification in infection control from the Certification Board of Infection Control and Epidemiology. Regarding implementation of EBP, the most frequently targeted pathogens were ESBL–producing Enterobacterales (87.3%), VRE (85.9%), MRSA (84.5%), and drug-resistant Streptococcus pneumoniae (84.5%). Some methods of initial staff education included posted guidelines/signage (83.3%), structured in-person sessions (77.4%), and informal staff meetings and discussions (72.6%). Facilities mainly used signage outside the resident’s door (93%), documentation in the resident’s care plan (79.1%), and EHR alerts (77.9%) to indicate EBP. When assessing perceptions of EBP, most respondents (47.3%) reported a somewhat or very negative perception of staff attitudes towards EBP, while (33.3%) reported a somewhat or very positive perception. Over half of respondents (58.3%) believed that EBP increases the overall cost of care in their facility. **Conclusion:** These findings provide important insight into real-world EBP implementation in nursing homes and identify perceived barriers and facilitators to adoption. Understanding infection preventionist perspectives is essential to refining EBP guidance, improving implementation strategies, and ensuring that MDRO prevention efforts in long-term care settings balance effectiveness with staff feasibility and resident-centered care.

